# Materials and devices for high‐density, high‐throughput micro‐electrocorticography arrays

**DOI:** 10.1016/j.fmre.2024.01.016

**Published:** 2024-02-28

**Authors:** Yang Xie, Yanxiu Peng, Jinhong Guo, Muyang Liu, Bozhen Zhang, Lan Yin, He Ding, Xing Sheng

**Affiliations:** a Department of Electronic Engineering, Beijing National Research Center for Information Science and Technology, Institute for Precision Medicine, Laboratory of Flexible Electronics Technology, IDG/McGovern Institute for Brain Research, Tsinghua University, Beijing 100084, China; b Beijing Engineering Research Center of Mixed Reality and Advanced Display, School of Optics and Photonics, Beijing Institute of Technology, Beijing 100081, China; c School of Materials Science and Engineering, The Key Laboratory of Advanced Materials of Ministry of Education, State Key Laboratory of New Ceramics and Fine Processing, Laboratory of Flexible Electronics Technology, Tsinghua University, Beijing 100084, China

**Keywords:** Electrocorticography, Micro-electrocorticography, Flexible electronics, Bioelectronics, Neural electrode array

## Abstract

The pursuit of precisely recording and localizing neural activities in brain cortical regions drives the development of advanced electrocorticography (ECoG) devices. Remarkable progress has led to the emergence of micro-ECoG (µECoG) devices with sub-millimeter resolutions. This review presents the current research status, development directions, potential innovations and applications of high-density, high-throughput µECoG devices. First, we summarize the challenges associated with accurately recording single or multiple neurons using existing µECoG devices, including passive multielectrode and active transistor arrays. Second, we focus on cutting-edge advancements in passive µECoG devices by discussing the design principles and fabrication strategies to optimize three key parameters: impedance, mechanical flexibility, and biocompatibility. Furthermore, recent findings highlight the need for further research and development in active transistor arrays, including silicon, metal oxide, and solution-gated transistors. These active transistor arrays have the potential to unlock the capabilities of high-density, high-throughput µECoG devices and overcome the limitations of passive multielectrode arrays. The review explores the potential innovations and applications of µECoG devices, showcasing their effectiveness for both brain science research and clinical applications.

## 1 Introduction

The pursuit of precise and efficient recording of neural electrical signals in the brain is crucial for advancing neuroscience research and facilitating clinical applications. The representative brain electrophysiological signals include scalp electroencephalography (EEG), stereotactic EEG (sEEG), electrocorticography (ECoG), etc. [1]. While scalp EEG is a non-invasive method that records brain electrical activity from the scalp using electrodes, it suffers from the limitations of high noise levels and low spatial resolution (∼cm) [2,3]. Concerns about the recording accuracy of EEG have constrained its applications in decoding signals during complex tasks [4]. sEEG involves the use of electrodes that penetrate the cortical layers within a stereotaxic framework to target specific deep brain structures for precise recording [5,6]. However, there are concerns about limited coverage of cortex and potential brain damage due to penetration [7,8]. ECoG measures signals from cortical neurons by placing electrodes directly on the exposed cortical surface, mainly consisting of low-frequency components (< 200 Hz) with amplitudes ranging from microvolts to millivolts [7,[9], [10], [11]]. Compared to EEG and sEEG, ECoG offers several advantages: (i) The ECoG technique captures neural electrical activity from the surface of the cerebral cortex, eliminating the need for electrode penetration and minimizing potential damage to brain tissue. (ii) It allows for high spatial resolution acquisition of signals from various brain regions and large coverage areas, enabling a comprehensive understanding of brain information. (iii) It exhibits tolerance for relative displacement between the brain and the device, contributing to its robustness. These features make ECoG a valuable tool for neuroscience research and clinical applications [12], [13], [14].

Conventional ECoG devices rely on large electrode contact sizes and wide spacing (millimeters to centimeters), resulting in a low electrode density (typically less than 0.1 sites/mm^2^) [1,15]. This design imposes constraints in the spatial resolution of mapping, making it challenging to localize specific brain regions [4,16]. However, recent advancements in flexible electronics and microelectronics have led to the development of micro-ECoG (µECoG) devices [8,17]. These devices utilize miniaturized electrodes (typically smaller than 1 mm²) and minimize interelectrode spacing, enabling higher spatial resolution and more precise measurement of brain spatial activity signal [8]. The emergence of µECoG presents opportunities for high-density, high-throughput ECoG devices, but it also introduces challenges of massive wiring and small size. Most µECoG devices rely on fixed wiring, which imposes constraints on electrode density due to complex lead connections when the number of channels increases [18]. Additionally, higher electrode density arrays result in smaller electrode contact sizes, leading to increased electrode impedance and lower signal-to-noise ratio (SNR). The dense placement of numerous electrodes in a small area also poses challenges in terms of mechanical flexibility and electrode-tissue contact. To ensure the biocompatibility of µECoG devices, optimization is necessary for material selection, packaging design, and other aspects. These optimizations aim to address challenges related to the mechanical properties of the device, the stability of electrode-tissue interfaces, and the long-term performance of the system.

**Fig. 1:**
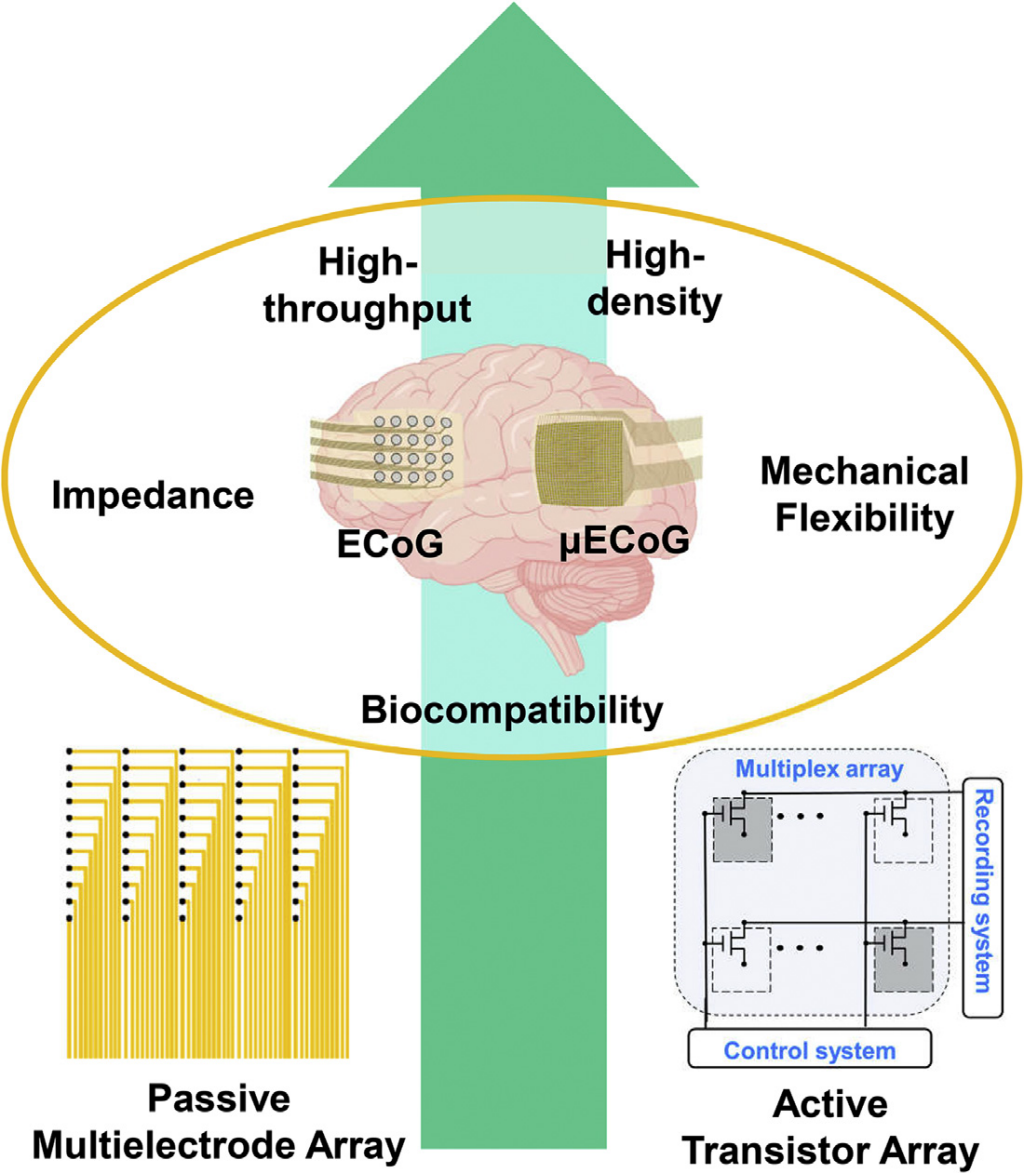
**Schematic of traditional ECoG devices, and high-density, high-throughput µECoG devices based on passive electrodes or active transistor arrays, as well as requirements for applications**.

This review specifically focuses on high-density, high-throughput µECoG devices and provides research status, development directions, potential innovations, and applications of µECoG devices (Fig. 1). The review considers two technological approaches: passive multi-electrode arrays and active transistor arrays. Passive devices have a long research history and have achieved significant progress through various technological methods. The paper emphasizes three key parameters for high-density, high-throughput µECoG devices: impedance, mechanical flexibility, and biocompatibility. It explores the design principles and fabrication strategies to optimize these parameters for passive multi-electrode arrays, while offering a comprehensive overview of cutting-edge advancements in the field. In the discussion of emerging active transistor arrays, the paper reviews several transistors that show promise in the roadmap for high-density, high-throughput µECoG devices.

## 2 Passive multielectrode arrays (MEAs)

Passive multielectrode arrays (MEA) are currently the predominant devices utilized for ECoG recording. Although comprehensive reviews have extensively covered passive MEA µECoG devices [8,17,[19], [20], [21]], this review takes a focused approach by selecting representative studies to explore optimization strategies for the crucial parameters of impedance, mechanical flexibility, and biocompatibility. These parameters are essential for achieving high-density and high-throughput µECoG devices. Representative works and device characteristics are also summarized in Table 1.

**Table 1:** **Representative works of µECoG arrays based on passive electrodes**.

Author	Year	Impedance	Electrode	Substrate	Subjects	Electrode size (µm)	Channels	Sensing area (mm^2^)	Density (sites/mm^2^)	Acute or Chronic
Y. Tchoe et al. [38]	2022	11 kΩ at 1 kHz	Pt Nanorods	Parylene C	Rat and human	Φ 30	1024/2048	23.04/6400	44.44/0.32	Acute
X. Li et al. [34]	2021	20.68 ± 6.65 kΩ at 1 kHz	MWCNTs/ PEDOT:PSS	PDMS-Parylene C	Rat	Φ 60	14	8.21	1.71	Acute
R. Dong et al. [43]	2021	250 ± 40 kΩ at 1 kHz	EGaIn	PDMS	Rat	Φ 100	16	9.73	1.64	Acute
T. Kaiju et al. [52]	2021	26 ± 7 kΩ at 1 kHz	Pt black	Parylene C	Monkey	50 × 50	1152	98	11.8	Acute
J.-W. Seo et al. [33]	2020	9.1 kΩ at 33 kHz	Au Nanonetwork	colorless PI	Mouse	∼Φ 200	16	6.76	2.37	Acute
A. C. Patil et al. [49]	2020	20 ± 1.21 kΩ at 1 kHz	Au	Silk	Rat	250 × 250	12	2.25	5.33	Acute
A. Schander et al. [32]	2019	1.1 ± 0.2 kΩ at 1 kHz	PEDOT	PI	Monkey	∼Φ 560	202	780	0.26	Chronic
M. Ganji et al. [37]	2019	16.89 ± 0.47 kΩ at 1 kHz	Pt Nanorods	Parylene C	Songbird, monkey and mouse	Φ 50	128	0.23	568	Acute
K. Tybrandt et al. [40]	2018	10 kΩ at 1 kHz	Au-TiO_2_ Nanowires	PDMS	Rat	50 × 50	32	∼0.85	38	Chronic
F. Vitale et al. [55]	2018	190 ± 72 kΩ at 1 kHz	Au/ECM	Parylene C/ECM	Rat	50 × 50	8	45	0.18	Chronic and acute
T. Kaiju et al. [52]	2017	11 ± 7.5 kΩ at 1 kHz	Au	Parylene C	Monkey	350	96	47.04	2.04	Acute
D. Khodagholy et al. [47]	2016	∼30 kΩ at 1 kHz	Planer PEDOT	Parylene C	Human	10 × 10	240	840	0.29	Acute
D. Khodagholy et al. [31]	2015	30 kΩ at 1 kHz	PEDOT	Parylene C	Rat and human	10 × 10	64	0.04	1600	Chronic and acute
D.-W. Park et al. [29]	2014	243.5 ± 5.9 kΩ at 1 kHz	Graphene	Parylene C	Rat and mouse	∼Φ 200	16	3.61	4.43	Chronic and acute
P. Ledochowitsch et al. [91]	2013	20∼30 kΩ at 1 kHz	Pt black	Parylene C	Rat	Φ 40	64	1.96	32.7	Chronic
H. Toda et al. [50]	2011	103 ± 5 kΩ at 1 kHz	Pt black	Parylene C	Rat	50 × 50	32	36	0.89	Chronic and acute
P. Ledochowitsch et al. [90]	2011	∼11 kΩ at 1 kHz	Pt	Parylene C	Rat	440 × 440	256	56	4.6	Acute
D.-H. Kim et al. [51]	2010	/	Au	PI/Silk	Cat	500 × 500	30	80	0.38	Acute

### 2.1 Electrode design strategies for low-impedance µECoG devices

Conventional MEA ECoG devices typically use flat electrodes made of noble metals as contacts. In this case, the electrical impedance *Z* at the electrode-electrolyte interface could be approximated by [22,23]

(1) $$\left|Z\right|=\;\frac{1}{A}\left(\frac{R}{\sqrt{1+\omega^{2}R^{2}C^{2}}}\right)$$

where *A* is the effective surface area of the electrode, *R* is the charge transfer resistance, *C* is the interface capacitance, and *ω* is the angular frequency of signals. Along with the size reduction of electrodes, achieving low-impedance contact electrodes with flat metal conductors becomes challenging in high-density µECoG arrays [18,[24], [25], [26]]. To address this challenge, researchers have explored the use of biocompatible materials with high conductivity, such as graphene, and conductive polymers like poly(3,4-ethylenedioxythiophene) (PEDOT). Graphene, in particular, has attracted considerable interest for neural electrodes owing to its exceptional properties, including high conductivity, mechanical flexibility, transparency, stability, and biocompatibility [27,28].

**Fig. 2:**
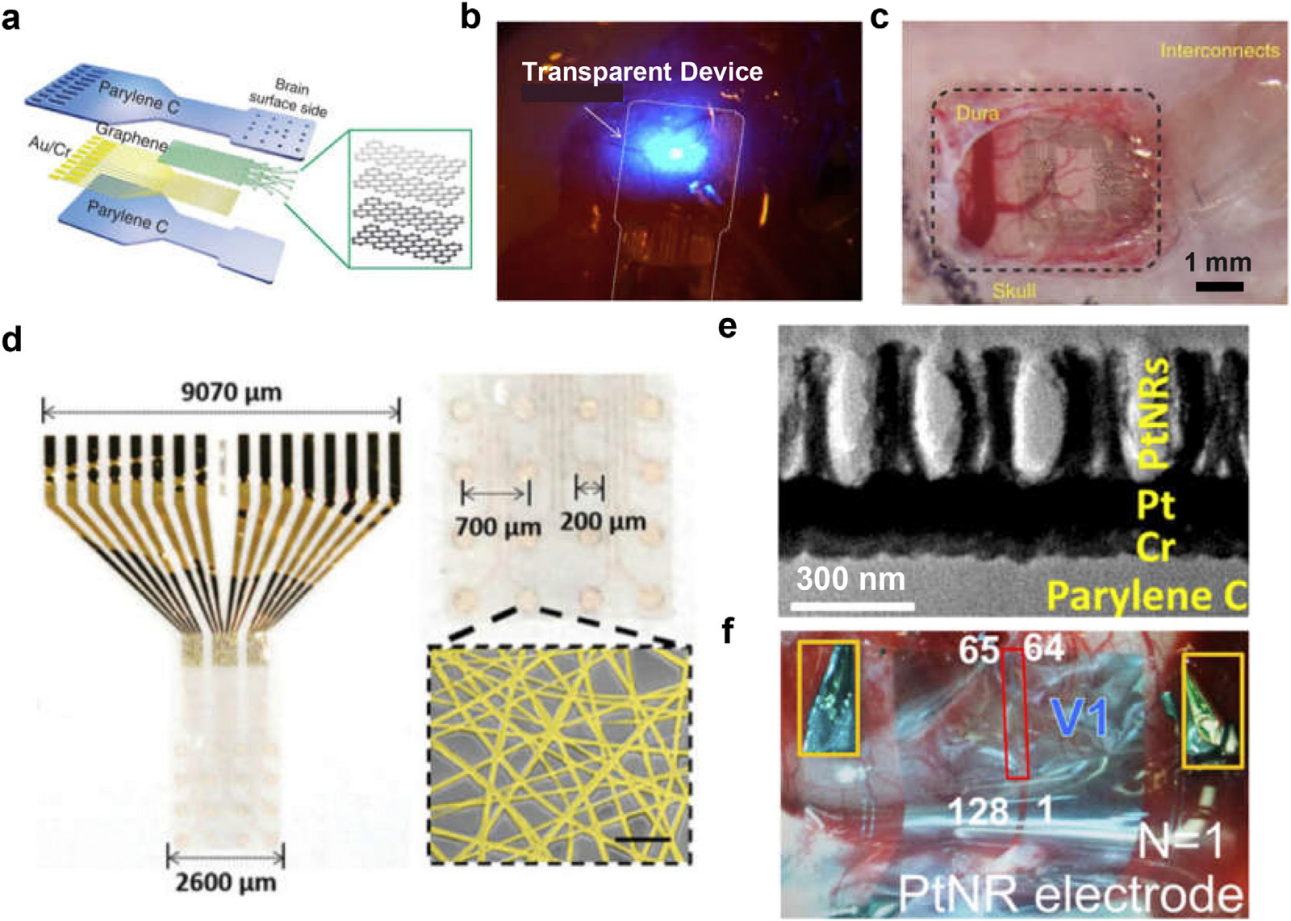
**Electrode design strategies for low-impedance µECoG devices**. (a) Schematic diagram and (b) Optical photograph of a transparent graphene-based MEA. Figures reproduced with permission from Ref. [29] Copyright (2014) Nature Publishing Group. (c) PEDOT-PSS based ECoG array. Figures reproduced with permission from Ref. [31] Copyright (2014) Nature Publishing Group. (d) High-transparency Au nanonetwork electrode array [33]. Copyright (2020) WILEY-VCH Verlag GmbH & Co. KGaA, Weinheim. (e) TEM of Pt nanorod based contact pads [37]. (f) 128-channel Pt nanorod based electrode positioned on the non-human primate cortex. Figures reproduced with permission from Ref. [37] Copyright (2019) American Chemical Society.

In a study conducted by D.-W. Park et al., a 16-channel µECoG device with planar graphene electrodes was developed as shown in Fig. 2a, achieving a maximum electrode density of 4.43 sites/mm² [29]. Transparent Parylene C was used as the support and encapsulation layers, allowing light transmission for optogenetics (Fig. 2b). Conductive polymers, such as PEDOT, offer a promising alternative for contact electrodes in high-density µECoG devices. PEDOT exhibits mixed electronic and ionic conductivity, facilitating a reduced electrochemical impedance between the tissue and electrodes due to its high ion migration rate [30]. Khodagholy et al. developed a high-density electrode with 64 channels, achieving a density of up to 64 sites/mm² by utilizing PEDOT as an interface material [31]. The electrode exhibited an impedance of approximately 30 kΩ at 1 kHz with a surface area of 10 × 10 µm² and a spacing of 30 µm, matching the average size and density of neurons in the brain (Fig. 2c). A. Schander et al. designed a 202-channel electrode with low impedance at 1 kHz (1.1 ± 0.2 kΩ) by leveraging the high conductivity of PEDOT as an interface material and employing a relatively larger electrode size of 0.25 mm² [32]. These studies highlight the effectiveness of PEDOT as a contact electrode material in achieving high-density µECoG devices with improved impedance characteristics.

Secondly, another approach to reducing electrode impedance and improving neuronal recording quality in high-density µECoG electrodes is surface modification. This optimization of impedance characteristics can be achieved by utilizing porous materials such as platinum black, noble metal nanomaterials, and carbon nanotubes (CNTs) to increase the effective surface area of the electrode [18,26]. In a study conducted by J.-W. Seo et al. a nanostructured electrode-based µECoG device with 16 channels and an electrode density of 2.37 sites/mm² was developed (Fig. 2d) [33]. Electrospun PMMA nano-fiber networks were utilized as a mask for nanopatterning an Au thin film, resulting in the formation of an Au Nanonetwork on the electrode surface. The Au Nanonetwork exhibited lower electrochemical impedance (8.1 kΩ at 19 kHz) compared to the Au film (8.1 kΩ at 11 kHz) due to its increased specific surface area. The nanostructure also enhanced the mechanical flexibility of the device, as evidenced by minimal degradation in bending performance after 30,000 cycles. Additionally, the Au Nanonetwork electrode displayed exceptional transparency, achieving a high transmittance of 75% on a transparent, colorless polyimide substrate. Another promising approach for impedance reduction in µECoG electrodes involves combining PEDOT and CNTs. X. Li et al. demonstrated the feasibility of this approach by developing a 14-channel µECoG array on a polydimethylsiloxane (PDMS)-parylene hybrid substrate [34]. The electrodes were modified with multiwalled carbon nanotubes (MWCNTs)/PEDOT:PSS nanocomposites through electrodeposition on the Au electrode surface. The nanocomposite-modified electrode demonstrated a substantial decrease in average impedance (20.2 ± 7.9 kΩ) compared to the bare gold microelectrode (392.2 ± 82.4 kΩ).

Furthermore, the incorporation of three-dimensional (3D) structures into electrodes has been investigated as an alternative approach to further enhance their impedance characteristics. For instance, nanorods can serve as templates for constructing a highly micrometer-scale array of conductive nanowires [35]. Another method involves using etching techniques to create porous conductive electrodes, effectively increasing their specific surface area [36], [37], [38]. M. Ganji et al. deposited Pt/Ag alloy onto a parylene C substrate and selectively dissolved the Ag, forming a platinum nanorod array on the electrode surface (Fig. 2e) [37]. Compared to planar Pt electrodes, this one-dimensional porous structure significantly increases the electrode's specific surface area, leading to reduced impedance (16.89 ± 0.47 kΩ at 1 kHz, one-tenth of the planar electrode) and enhanced charge injection capacity (4.4 mC·cm^–2^, 16 times higher than the planar electrode). Based on this strategy, the authors designed and fabricated a high-density µECoG device with 128 channels, enabling the recording of visual cortical signals in non-human primates (Fig. 2f). This advancement highlights the potential of incorporating 3D electrode structures to improve the performance of µECoG devices.

### 2.2 µECoG devices with designed mechanical flexibility

In the pursuit of enhancing µECoG throughput and density, prioritizing mechanical flexibility is crucial. This emphasis ensures improved mechanical and physical compatibility between the µECoG device and brain tissue, facilitating the establishment of stable and reliable ohmic contact between the electrodes and the tissue [17,21,39]. As a result, it leads to enhanced signal recording quality and plays a significant role in reducing inflammation responses in the brain tissue. An effective strategy to enhance the mechanical flexibility of µECoG devices is the utilization of stretchable materials with a low elastic modulus. By incorporating such materials into the design of recording devices, it becomes more compliant and capable of accommodating the natural movements and contours of the brain. This promotes a better interface between the electrodes and the brain tissue, allowing for more accurate and reliable signal recordings. Additionally, the use of stretchable materials helps minimize the risk of tissue damage or irritation, further contributing to the overall performance and biocompatibility of the µECoG device [17,39].

**Fig. 3:**
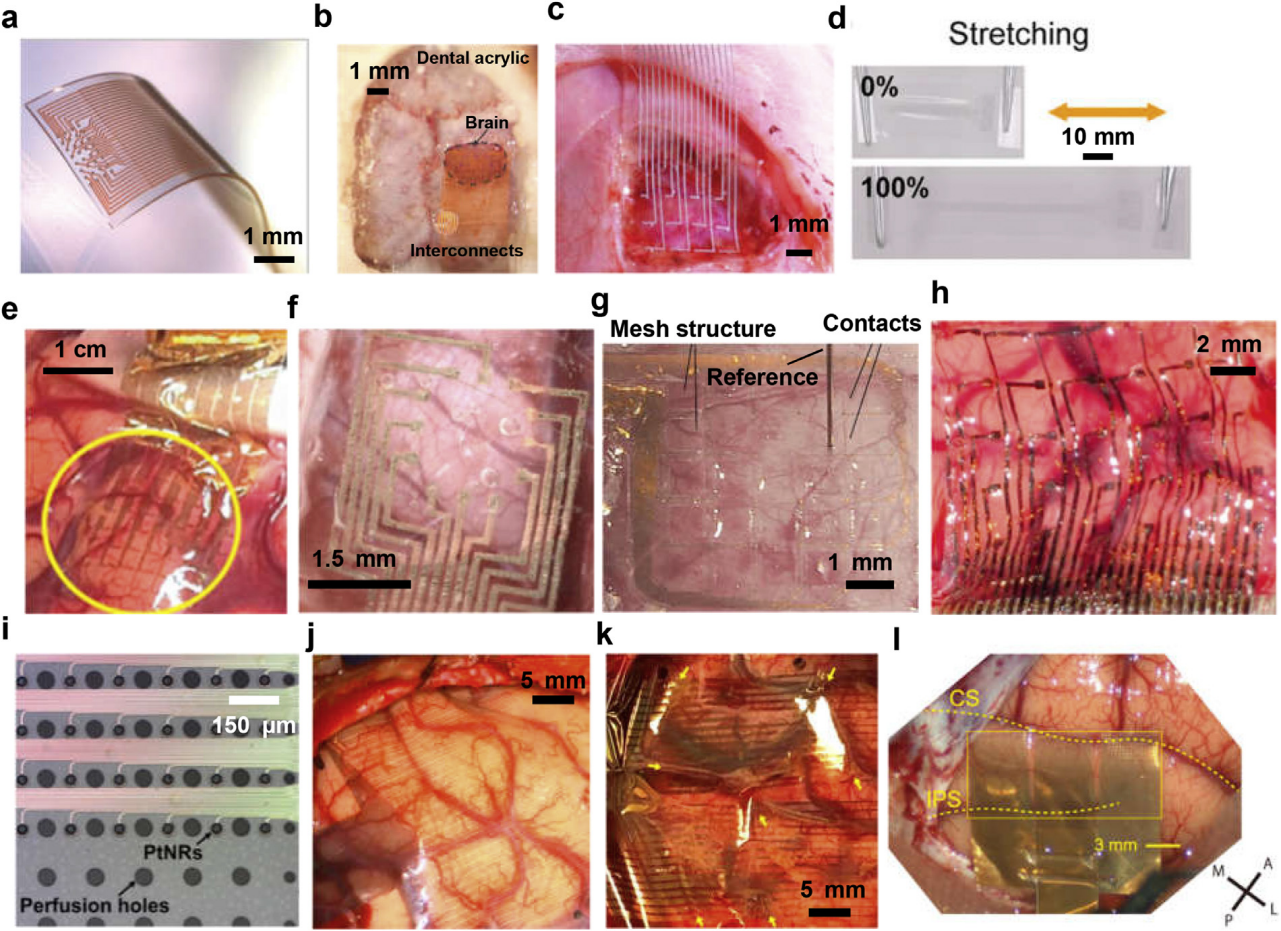
**µECoG devices with designed mechanical flexibility**. (a) Optical micrograph of a freestanding ECoG device based on PDMS substrate and (b) placed on surface of a rat brain. Figures reproduced with permission from Ref. [40]. Copyright (2018) Nature Publishing Group. (c) Microphotograph of the 16-channel liquid-metal electrodes on a rat brain. (d) Snapshots of liquid metal neural electrodes after being stretched with 0% and 100% strain. Figures reproduced with permission from Ref. [43]. Copyright (2021) Wiley-VCH GmbH. (e) 4 µm-thick ultrathin ECoG devices placed on the human cortex. Figures reproduced with permission from Ref. [47] Copyright (2016) Nature Publishing Group. (f) ECoG device on 15 µm-thick silk substrate. Figures reproduced with permission from Ref. [49] Copyright (2019) Elsevier Ltd. (g) ECoG devices utilizing a mesh-structured Parylene-C substrate fabricated through plasma etching. Figures reproduced with permission from Ref. [50] Copyright (2011) Elsevier. (h) Mesh structure formed by bioresorbable silk substrate. Figures reproduced with permission from Ref. [51] Copyright (2010) Nature Publishing Group. (i) Photograph of a high-density ECoG array with perfusion holes. Figures reproduced with permission from Ref. [38]. Copyright (2022) American Association for the Advancement of Science. (j) Devices with and (k) without perfusion holes on top of the human cortex. Figures reproduced with permission from Ref. [38] Copyright (2022) American Association for the Advancement of Science. (l) A 1152-channel ECoG device consisting of a comb-like structure formed by combining nine 8 × 16 electrode arrays in a 3 × 3 design. Figures reproduced with permission from Ref. [52] Copyright (2021) The Author(s).

K. Tybrandt et al. designed and fabricated of a 32-channel electrode array utilizing PDMS as the base material as illustrated in Fig. 3a and 3b [40]. The device incorporated Au-TiO_2_ nanowire composites for conductive interfaces. The porous nature of this material enhances the mechanical properties of the conductive layer and reduces the contact impedance to 10 kΩ at 1 kHz by increasing the effective surface area of electrodes. Notably, the device exhibited excellent mechanical flexibility, maintaining stability even after undergoing 1000 cycles of 100% strain. After a three-month implantation in rat brains, the majority of electrodes continued to function reliably. In the realm of organic electrode materials, such as polypyrrole (PPy), they demonstrated excellent compatibility with stretchable microsystems. D. Qi et al. successfully designed and fabricated µECoG devices capable of accommodating up to 110% strain using PPy electrode material and a PDMS substrate [41]. Liquid metals also hold promise as viable options for flexible µECoG devices due to their exceptional mechanical properties and conductivity [42]. R. Dong et al. developed a flexible µECoG electrode array (Fig. 3c) with 16 channels and a density of approximately 1.64 sites/mm², utilizing a eutectic gallium-indium alloy (EGaIn) [43]. The contact electrodes, modified with Pt, exhibited an impedance of 250 ± 40 kΩ. The device maintained stable electrical and mechanical performance even under strains exceeding 100% (Fig. 3d). Recently, Q. Zhuang et al. introduced a novel fabrication process for wafer-scale liquid metal microelectrodes, allowing for electrode density of up to 755 sites/mm² [44]. They further demonstrated the feasibility of this approach by fabricating a 36-channel µECoG device with a density of 1 sites/mm² for animal experiments.

However, the brain has Young's modulus of only around 1 kPa, which is incompatible with most commonly used flexible materials (e.g., PI with approximately 8 GPa, Parylene C with 2.8 GPa, PET with 4 GPa) [45]. In addition to utilizing low-modulus materials, another approach is to enhance the contact between high-density µECoG devices and the intricate surface of the cerebral cortex to reduce the device thickness [17,21,46]. For instance, Khodagholy et al. developed an ultra-thin parylene-based microelectrode array (MEA) with a thickness of merely 4 µm for human brain ECoG recording. The ultra-thin substrate enabled the device to tightly conform to the complex structures of the human brain, covering an area of 840 mm^2^, and acquiring stable signals from 240 channels (Fig. 3e) [47]. Silk fibroin, known for its excellent biocompatibility and mechanical properties, can also be utilized as a substrate for ultra-thin µECoG devices [48]. A. C. Patil et al. fabricated a non-transient water-sTable 12-channel µECoG array by encapsulating gold electrodes within a double-layered 15 µm silk substrate. The thin design, combined with the intrinsic characteristics of silk fibroin, facilitated stable and conformal adhesion to the target tissues (Fig. 3f) [49].

Another approach to improve device-tissue contact involves designing the geometric structure of the device. H. Toda et al. fabricated a 32-channel mesh electrode array (Fig. 3g) with dimensions of 6 mm × 6 mm and a 1 mm spacing between electrodes, and the gold electrode surfaces were modified with platinum black to enhance impedance characteristics [50]. When implanted on the dura/meninges surface of the rat visual cortex for up to two weeks, the mesh electrode array exhibited stable impedance without significant changes, enabling proper ECoG signal recording. Utilizing degradable matrices is another ingenious approach to attaching prefabricated mesh µECoG electrodes to cortical surfaces. D.-H. Kim et al. employed silk films as temporary substrates, which degraded after adhering the device to the cortical surface, resulting in a mesh electrode array with a 30-channel spacing of 2 mm (Fig. 3h) [51]. The device maintained conformal contact with the feline cortical surface, allowing for the detection of high-quality sleep spindles in ECoG recordings, thus demonstrating the feasibility of this approach.

Creating perforations in the gaps between electrodes can be a simpler solution to achieve tighter coverage in certain contexts. In a recent study by Youngbin Tchoe et al., they developed a high-density electrode array with a thousand channels and a density of up to 26 sites/mm² [38]. The device utilized Parylene C as the substrate and encapsulation layer, resulting in a total thickness of less than 7 µm and excellent mechanical flexibility. This design allowed for high spatial resolution and cortical coverage, facilitating detailed brain mapping and studying disease areas. To optimize the device-brain tissue interface, perforation patterns (Fig. 3i) were introduced in the blank areas between microelectrodes on the device substrate. These perforations facilitated the infusion of saline and cerebrospinal fluid during the surgical procedure, resulting in closer adherence of the device to the cortical surface (Fig. 3j) compared to devices without infusion holes. This closer adherence enhances contact and improves the overall performance of the device. Another approach to enhancing the mechanical flexibility of high-density µECoG devices is by utilizing a comb-like structure [52]. T. Kaiju et al. developed an 1152-channel µECoG device that covered a larger area (14 mm × 7 mm). The device consisted of nine 8 × 16 grid MEA electrode arrays arranged in a comb-like stru cture (3 × 3 configuration), which facilitated interconnections and promoted better adherence to the cortical surface (Fig. 3l). This comb-like structure improves the conformal contact of the device with the brain tissue, leading to more reliable signal recordings and improved overall performance.

### 2.3 Biocompatibility of µECoG devices

Traditional ECoG devices have undoubtedly made significant strides in clinical applications, showcasing their long-term feasibility [53,54]. However, the persistent challenge of the foreign body response from neural tissue to implanted ECoG electrodes remains a considerable barrier [19]. This concern becomes even more pronounced for emerging high-density, high-throughput µECoG devices, as innovative design approaches increasingly prioritize biocompatibility. Therefore, optimizing the biocompatibility of high-density µECoG devices is a promising endeavor to address immune responses and improve long-term implantation outcomes. One strategy is the application of biocompatible coatings to the devices, which can enhance interface properties and reduce immune reactions. For example, F. Vitale et al. modified an 8-channel µECoG device with a microscale extracellular matrix hydrogel, which effectively reduced chronic foreign body response without compromising the device's recording capabilities [55]. This approach was validated in a rat model, demonstrating its potential for improving biocompatibility. In addition to coatings, the use of degradable materials holds significant potential for enhancing biocompatibility in µECoG devices. Degradable materials offer the advantage of controlled release of anti-inflammatory biomolecules or healing drugs and enable the development of transient electronic devices. Various biodegradable materials are commonly used in ECoG devices, including carbohydrates, carboxymethyl cellulose (CMC), silk protein, alginate, polylactic acid (PLA), polyethylene glycol (PEG), and poly(lactic-co-glycolic acid) (PLGA). These materials can be combined with degradable conductive materials such as zinc (Zn), magnesium (Mg), molybdenum (Mo), or heavily doped silicon (Si) to fabricate degradable devices [19,56]. For example, K. J. Yu et al. proposed a silicon nanomembrane-based biodegradable µECoG device that dissolves in biological fluids [57]. This flexible device, with its ultrathin structure, enables conformal contact with the cortical surface of the brain and facilitates ECoG signal recording. The authors demonstrated the potential of this approach by fabricating a 256-channel microelectrode array (MEA) device, highlighting the feasibility of using degradable materials for high-density µECoG applications.

Despite the remarkable progress achieved in MEA-based µECoG devices, the use of fixed wiring connections inevitably results in spatial redundancy in device design. Typically, these devices have a limited number of contact points, while the wiring occupies a significant amount of space. Researchers have explored two approaches to improve the electrode density of µECoG devices: densely arranging contact points in smaller spaces using finer wiring or implementing a multi-layer structure for high-density MEA contact point external connections. However, both approaches require the placement of bulky wiring, leading to a larger coverage area and posing challenges in connecting numerous wires between the electrode array and the data acquisition system. As the channel count and density of µECoG devices continue to increase, the continued use of fixed wiring methods becomes increasingly challenging.

## 3 Active transistor array

Active transistor arrays with multiplexing capabilities and improved sensitivities due to in-situ amplification, stands as a crucial avenue in the advancement of high-density µECoG devices. In the subsequent discussion, we overview active devices based on the following materials: silicon, metal oxide, and solution-gated transistor arrays, and highlight their biomedical applications. Representative works and device characteristics are also summarized in Table 2.

**Table 2:** **Representative works of µECoG arrays based on active transistors**.

Author	Year	Impedance	Devices	Electrode	Substrate	Subjects	Electrode size (µm)	Channels	Sensing area (mm^2^)	Density (sites/mm^2^)	Acute or Chronic
E.T. Zhao et al. [59]	2023	/	CMOS	Pt	PI	Mouse	Φ 20	504	∼0.58	873	Acute
M. Wu et al. [75]	2023	/	OECT	PEDOT:PSS (channel)	PLGA	Rat	200 × 20 (channel size)	100	64	1.56	Acute
X. Huang et al. [69]	2022	16.5 ± 2.3 kΩ	IGZO	Au	PI	Mouse	Φ 300	256	640	4	Acute
C.-H. Chiang et al. [64]	2020	∼230 kΩ at 1 kHz	Si	Au/SiO_2_ (capacitive sensing)	PI	Monkey	100 × 180	1008	83.16	12.1	Chronic and Active
N. Schaefer et al. [83]	2020	/	Graphene	Graphene (channel)	PI	Rat	50 × 50	64	6.55	9.77	Acute
R. Garcia-Cortadella et al. [84]	2020	/	Graphene	Graphene (channel)	PI	Rat	50 × 50	32	3.36	9.52	Acute
W. Lee et al. [74]	2017	/	OECT	PEDOT:PSS (channel)	Parylene C	Mouse	90 × 60 (channel size)	15	15	1	Acute
M.A. Escabí et al. [61]	2014	∼45 kΩ at 1 kHz	Si	Pt	PI	Rat	200 × 200	196	12.25	16	Acute
J. Viventi et al. [60]	2011	∼20 kΩ at 1 kHz	Si	Pt	PI	Cat	300 × 300	360	90	3.6	Acute

### 3.1 Silicon-based transistor array for ECoG recording

Multiplexing technology refers to the technique of combining multiple low-speed channels into a single high-speed channel. In such active arrays, transistors act as switches to control the signal transmission of electrophysiological sensors, achieving multiplexed transmission of multiple sensing signals on a single channel through time-division multiplexing. Additionally, these active transistors are often employed as electrophysiological sensors, for instance, as the gate electrode of a transistor capable of in situ amplification of neural signals. With fixed wiring technology, routing 10,000 electrodes and wires would be necessary. By contrast, with multiplexing technology, a minimum of only 200 wires (100 × 100 array, 100 row selector wires and 100 column recording wires) would be required. This significant reduction in the number of wires not only reduces the space required for wiring but also alleviates the difficulties associated with wire routing. Therefore, multiplexing technology plays an indispensable key role in the development of future high-density µECoG devices. Silicon-based complementary metal-oxide-semiconductor (CMOS) sensors with multiplexing capabilities present promising solutions to realizing high-density, high-throughput ECoG devices. The recently developed Argo system connects microwire electrode arrays with silicon-based CMOS sensors, enabling the recording of LFP signals with a high spatial resolution [58]. Additionally, the CMOS sensor is interfaced with flexible ECoG devices containing thousands of channels for in vitro validation and performed seizure recordings in awake and behaving mice using a device with 504 channels [59].

**Fig. 4:**
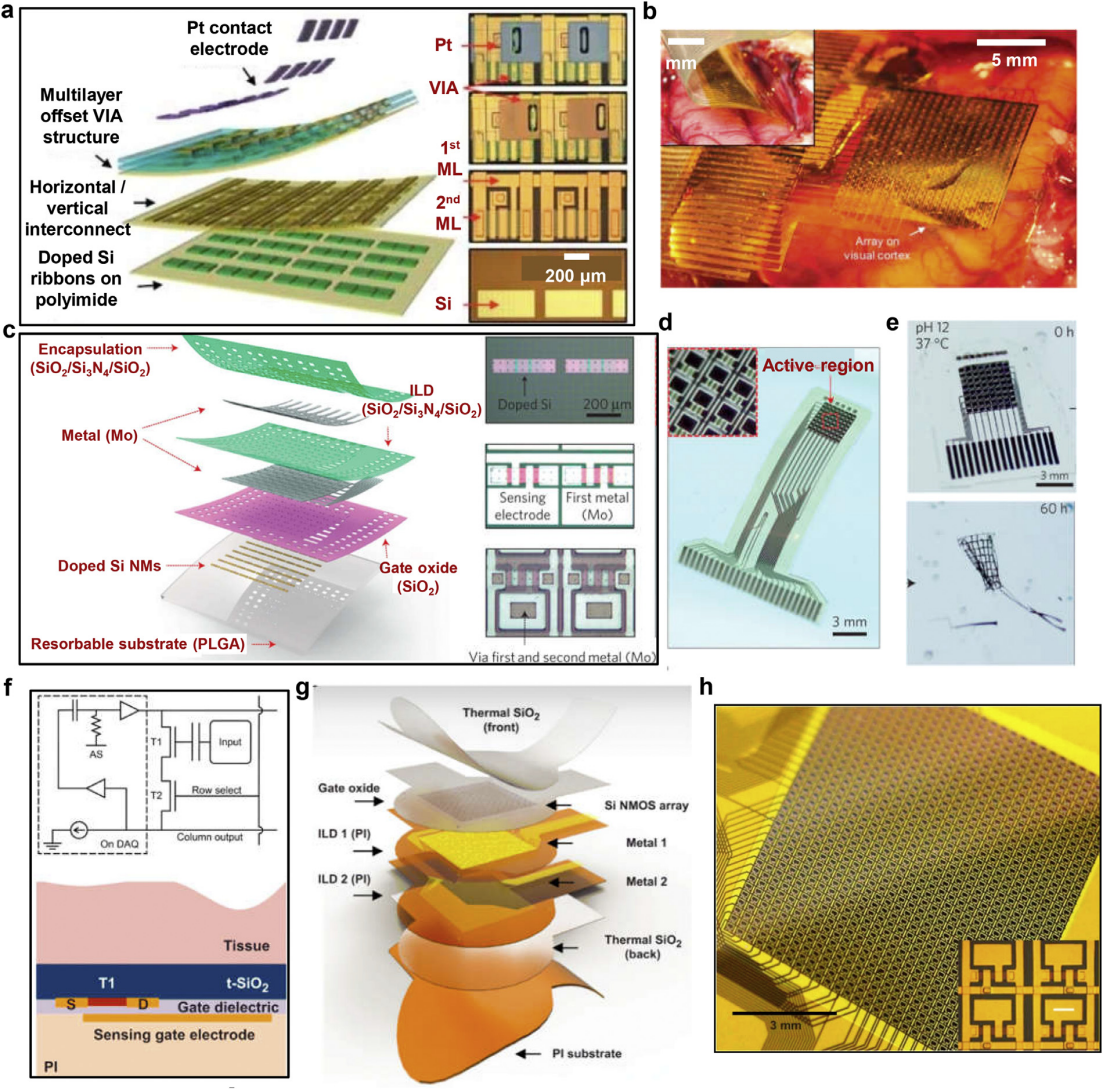
**Silicon-based transistor array for ECoG recording**. (a) Exploded illustration of a 360-channel active multiplexed ECoG devices based on silicon membrane transistors. (b) The flexible high-density silicon transistor array placed on the visual cortex of a rat. Figures reproduced with permission from Ref. [60] Copyright (2011) Nature Publishing Group. (c) Exploded illustration of a fully biodegradable multiplexed silicon-based transistor array. (d) Optical micrographs of the complete biodegradable device system. (e) Images captured during the accelerated dissolution process of a system submerged in a buffer solution (pH = 12) at 37 °C, demonstrating the biodegradability of the device. Figures reproduced with permission from Ref. [57] Copyright (2016) Nature Publishing Group. (f) Schematic diagrams of the capacitive sensing transistor array. (g) Exploded-view illustration of a silicon-based transistor devices designed for long-term ECoG recording. (h) Photos of a 1008-channel capacitive sensing array. Figures reproduced with permission from Ref. [64] Copyright (2022) American Association for the Advancement of Science.

Another pathway to achieve multiplexing is through the use of silicon thin-film transistor arrays. Viventi et al. have developed a novel 360-channel active multiplexed device with ultrathin and flexible silicon membrane transistors integrated onto a nonpenetrating electrode array [60]. Fig. 4a illustrates the fabrication process of ultrathin silicon transistors (10–500 nm), which were made from high-quality single-crystal silicon. These transistors exhibited an on/off ratio greater than 10^3^ and mobility of approximately 350 cm^2^ V^−1^. A total of 720 silicon membrane transistors were integrated onto a thin polyimide substrate (12.5 µm), resulting in an active sensing coverage of approximately 100 mm^2^. Each recording site had dimensions of 300 × 300 µm^2^ with a spacing of 500 µm and demonstrated an impedance of 18 Ω cm^−2^ at 1 kHz. The array's single unit cell consisted of two matched transistors: one serving as an electrophysiology sensor by monitoring gate voltage changes, and the other acting as a switch for multiplexing purposes. This strategy allowed for in-situ amplification, enhancing sensitivity but increasing the unit area [61]. With only 38 interconnection lines for fast addressing, the device successfully recorded high-density µECoG signals (360 channels) from adult cats (Fig. 4b). It offered high spatial resolution with a spacing of 500 µm and high temporal resolution with a sampling rate of approximately 277 Hz per active electrode.

The biodegradable properties of silicon open new avenues for multiplexed ECoG devices. K. J. Yu et al. developed a biodegradable silicon array that allows for natural degradation following high-resolution brain area recording [57]. The device is based on single-crystal silicon thin-film transistors and utilizes biodegradable PLGA as the substrate. Metal molybdenum (Mo) is used for interconnects and sensing electrodes, while SiO_2_ and Si_3_N_4_ serve as encapsulation and dielectric layers. This configuration results in a fully biodegradable multiplexed µECoG device (Fig. 4c). The device encompasses 64 recording channels within an active area of approximately 12 mm^2^, requiring only 26 lead wires (Fig. 4d). Accelerated dissolution experiments conducted in a pH 12 PBS solution at 37 °C demonstrate its biodegradability within biological tissues (Fig. 4e). The instability of silicon materials in living organisms has posed challenges for long-term recording using silicon transistor arrays. In subsequent studies, H. Fang et al. introduced silicon dioxide as a sensing interface, enabling long-term stable recording of in vivo electrophysiological signals through capacitive coupling sensing [62]. The capacitive coupling sensing array incorporates a silicon dioxide film at the bottom of the sensing electrode, forming a tissue/SiO_2_ dielectric layer/gate dielectric layer/gate sensing electrode capacitor. This design allows for capacitive coupling sensing of electrophysiological signals, and the reliable SiO_2_ encapsulation layer significantly enhances the device's long-term stability (Fig. 4f) [63,64]. C.-H. Chiang et al. designed and fabricated a µECoG device with 1008 channels (28 × 36 rectangular array, electrode size of 100 µm × 180 µm, electrode spacing of 330 µm) based on silicon thin-film transistors and capacitive coupling sensing [64]. The device covers approximately 1 cm^2^ of recording area and demonstrated long-term recording capabilities in rats (over 1 year) as well as high-density, multi-brain region recording in non-human primates (NHPs), achieving an effective final sampling rate of 781.25 Hz per channel (Fig. 4g, 4h). Recently, K.J. Seo, et al. developed a filamentous, flexible silicon-based transistor array with 256 channels and effectively demonstrated its capability to record neural signals through acute auditory experiments in rats [65]. Silicon membrane transistor-based µECoG devices have indeed made significant progress and are considered a reliable option for multiplexed µECoG systems. However, there are still challenges associated with achieving extensive integration of single-crystal silicon TFTs on flexible substrates. The complex nature of this task presents difficulties in terms of fabrication. Additionally, the higher costs associated with the fabrication process limit the broader utilization of silicon TFT-based µECoG devices. These factors need to be addressed in order to overcome the challenges and enable wider adoption of these devices.

### 3.2 Multiplexed µECoG array made of metal oxide based thin-film transistors

Metal oxide-based thin-film transistors (TFTs) have been extensively used in optoelectronic displays and offer several advantages over silicon-based materials. They can be directly fabricated on large flexible substrates, providing flexibility in processing and cost-effectiveness. The precise fabrication processes and operating frequency parameters of metal oxide TFTs meet the requirements for ECoG signal acquisition, offering the potential to overcome current technological limitations and achieve a new high-density, flexible ECoG recording scheme with ultra-high throughput.

**Fig. 5:**
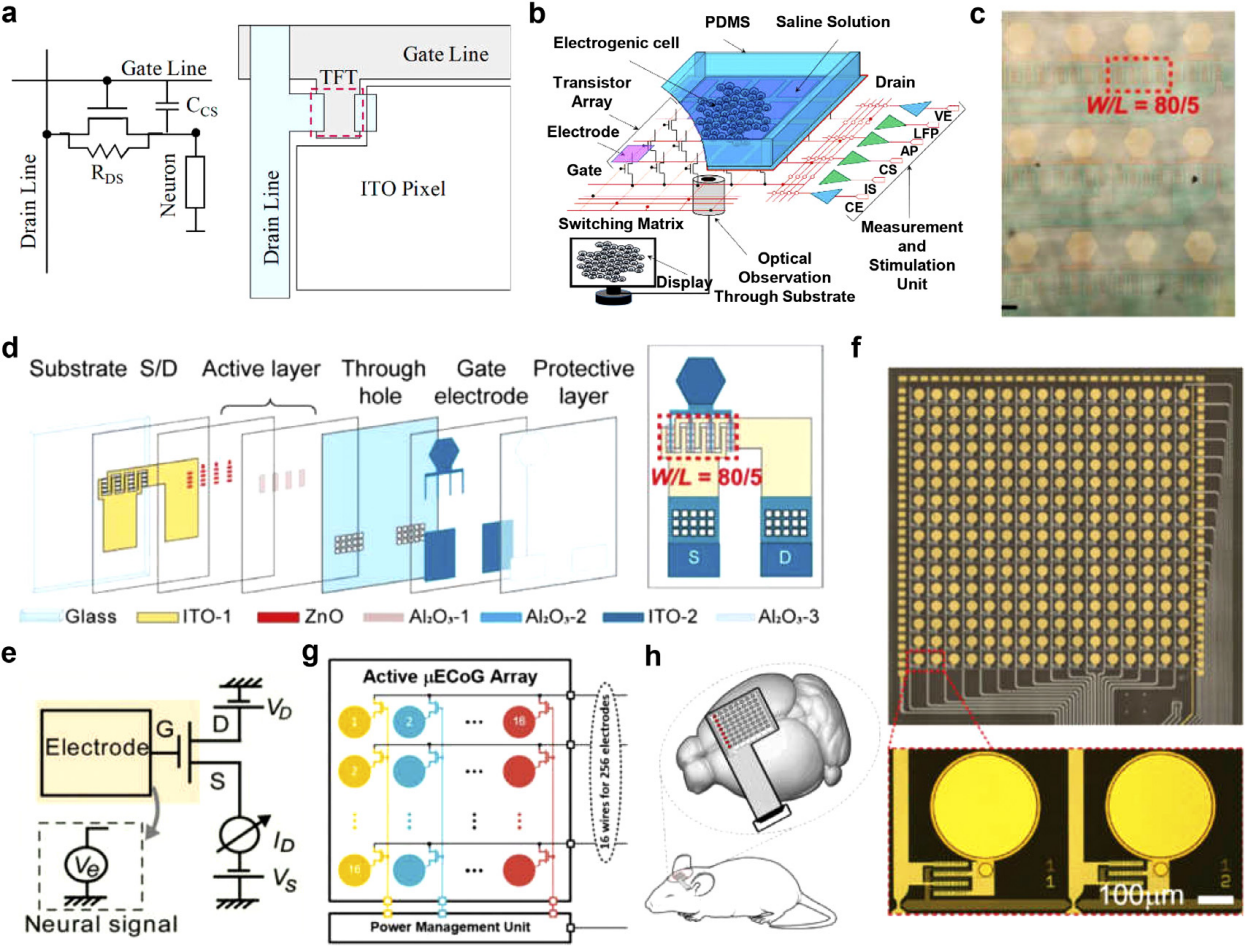
**Multiplexed µECoG array made of metal oxide based thin-film transistors (TFTs)**. (a) Schematic illustration of a pixelated array and electrical switching system in a TFT biosensor chip [66]. Copyright (2020) Elsevier B.V. (b) The operation scheme of the TFT multiplexed array. Figures reproduced with permission from Ref. [66] Copyright (2022) Elsevier B.V. (c) Optical micrograph and (d) Schematic diagram of the structure of an active electrode array based on ZnO TFTs. Figures reproduced with permission from Ref. [67] Copyright (2022), Wiley-VCH GmbH. (e) Circuit diagram of a ZnO-TFT electrode array [67]. Copyright (2022) Wiley-VCH GmbH. (f) Circuit diagram and (g) The photograph of a 256-channel active multiplexed IGZO-TFT µECoG array [68]. Copyright (2022) Institute of Electrical and Electronics Engineers. (h) Setup illustration of an 8 × 8 IGZO-TFT array utilized for ECoG recording from the mouse brain, emphasizing the configuration of eight multiplexed electrodes sharing a common super-channel [69]. Copyright (2022) Institute of Electrical and Electronics Engineers.

Rigid TFT arrays based on glass substrates have been utilized for recording cellular potentials. In a study by F. A. Shaik et al., a configuration of indium gallium zinc oxide (IGZO) TFTs and indium tin oxide (ITO) electrodes were implemented within a sensing area, with varying electrode spacing (Fig. 5a) [66]. IGZO is a prevalent channel material in TFTs, offering desirable carrier mobility and optical transparency. ITO possesses ideal conductivity and high optical transparency, serving as a common transparent electrode. In this study, IGZO acts as the channel active layer in TFTs. Meanwhile, ITO serves as the contact electrode to interact with neurons and also acts as the conductor for the source, gate, and drain in the TFT configuration. The TFTs function as a switch matrix, enabling different recording and stimulation modes (Fig. 5b). The device successfully recorded neuronal spike signals at a sampling rate of 25 kS s^−1^, demonstrating the suitability of TFT arrays for biological measurements. More recently, metal-oxide semiconductor thin-film transistor arrays have been investigated for ECoG recording. F. Zhang et al. designed and fabricated a transparent zinc oxide (ZnO) TFT array, validating its capability to acquire ECoG signals using mice as an animal model (Fig. 5c) [67]. The device employed optically transparent designs and facilitated seamless integration with optogenetics applications (Fig. 5d). The operational principle involved collecting ECoG signals through gate sensing electrodes, where voltage variation at the gate electrode modulated the source-drain current, enabling ECoG signal reading (Fig. 5e). Flexible TFT arrays for ECoG recording have also gained attention. X. Huang et al. presented a scheme for active multiplexed µECoG recording using an IGZO TFT array (Fig. 5f, 5g) [68,69]. The acquisition principle involved the multiplexed acquisition of ECoG signals, but the presence of electrode DC offset posed challenges. Additionally, the array employs a drain electrode sensing design without in-situ amplification functionality. The schematic diagram of a biological experiment conducted in mice demonstrated the feasibility of this approach (Fig. 5h). Although challenges remain for the multiplexed acquisition of metal oxide TFTs in ECoG recording, their well-established flexible fabrication processes and exceptional switching characteristics position them as a highly promising pathway for achieving high-density, high-throughput ECoG recording schemes.

### 3.3 µECoG arrays based on solution-gated transistors

**Fig. 6:**
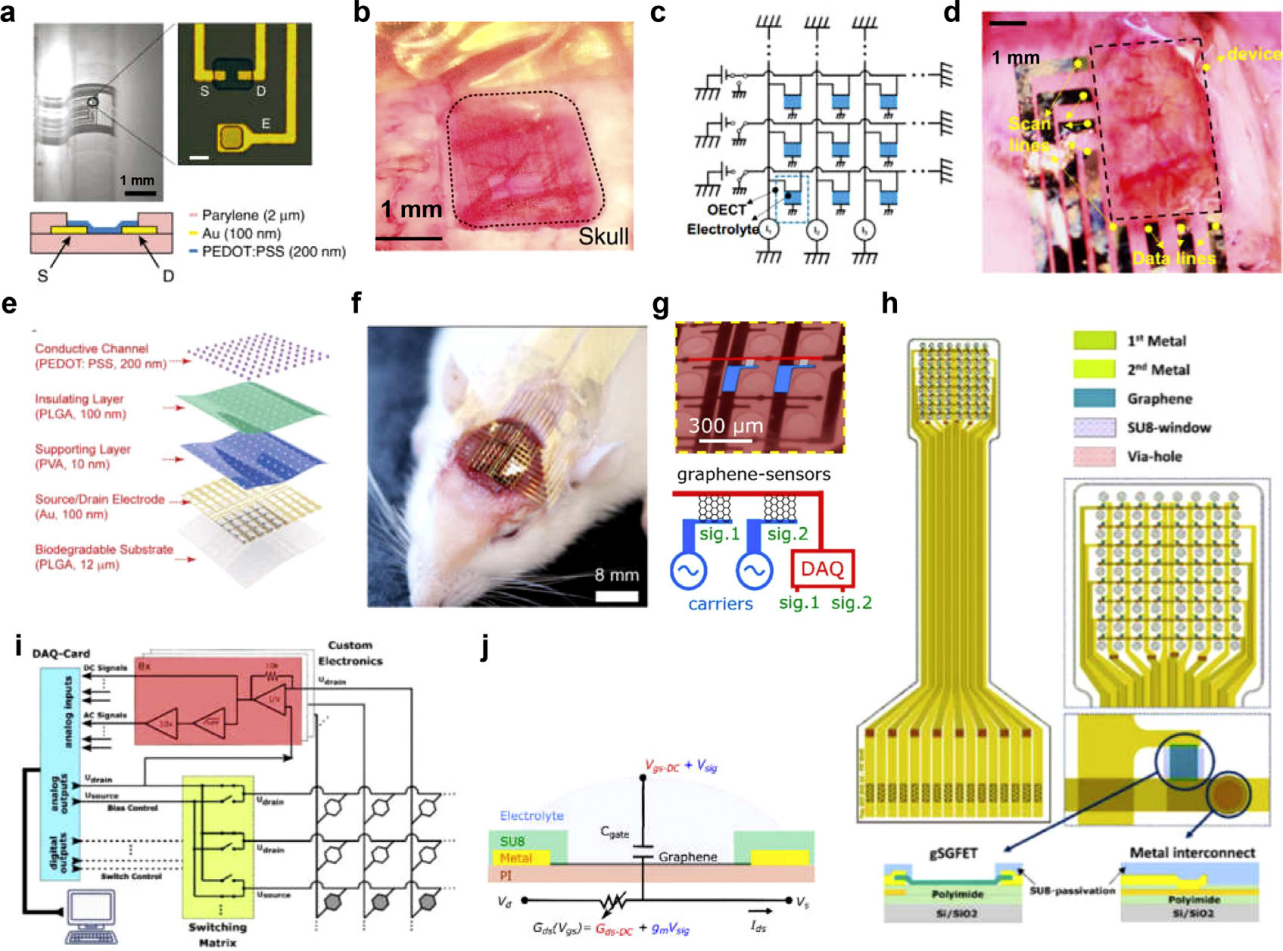
**µECoG arrays based on solution-gated transistors**. a. Optical micrograph of an OECT array, with the channel material made of PEDOT:PSS [73]. Copyright (2013) Nature Publishing Group. b. The OECT array positioned on the somatosensory cortex, with dashed lines indicating the region of the skull window [73]. Copyright (2013) Nature Publishing Group. c. Circuit diagram of the OECT array with current meters connected to drain and source lines under applied voltage, while other lines are grounded to avoid crosstalk [74]. Copyright (2017) PNAS. d. A 3 × 5 OECT array on a parylene substrate placed on the cortical surface of a rat (black dashed square) for ECoG recording during optogenetic stimulation [74]. Copyright (2017) PNAS. e. Exploded illustration of a fully biodegradable OECT array consisting of 100 units with an area of 8 × 8 mm² [75]. Copyright (2023) Wiley-VCH GmbH. f. Photograph of the OECT array seamlessly attached to the cerebral cortex of a rat [75]. Copyright (2023) Wiley-VCH GmbH. g. Microscopic image of 4 × 8 graphene-based neural probes implanted on the rat cortex, with the g-SGFET array connecting the brain carriers to a data acquisition system [83]. Copyright (2020) American Chemical Society. h. Layout and cross-sectional view of a flexible ECoG array with 64 g-SGFETs [83]. Copyright (2020) Institute Of Physics. i. Schematic of a discrete multiplexing setup featuring a DAQ-card for bias control and read-out (blue), accompanied by a custom-built PCB board for switching (yellow), filtering, and current-to-voltage conversion (red) [84]. Copyright (2020) American Chemical Society. j. Equivalent circuit and crosssection illustration of the g-SGFET device, showing the applied gate and drain voltage along with the measurement of drain-source current [84]. Copyright (2020) American Chemical Society.

Transistors based on silicon and metal oxide semiconductor have been successfully integrated into in vivo µECoG devices for simultaneous recording from multiple electrodes. However, these devices face challenges when used in living organisms, mainly due to the inadequate biocompatibility of the transistor oxide layer and the presence of a water-based environment in biological tissues [60]. To address these challenges, solution-gated transistor-based ECoG recordings incorporate organic electrochemical transistors (OECTs) or graphene solution-gated field-effect transistors (g-SGFETs), which offer promising solutions for in vivo electrophysiological recording of neuronal circuits. OECTs typically utilize thin-film conjugated semiconductors or conductive polymers as channels, such as poly(3,4-ethylenedioxythiophene) doped with polystyrene sulfonate (PEDOT:PSS) [70]. The OECT device consists of a channel with source and drain electrodes connected at both ends, and the surface of the film is in direct contact with the electrolyte solution. By applying a gate voltage to the electrolyte solution, ions from the electrolyte migrate into the organic semiconductor channel, modulating its conductivity and resulting in the modulation of the drain current [71,72]. Dion Khodagholy et al. developed an µECoG probe based on PEDOT:PSS OECT and a gate electrode, as shown in Fig. 6a [73]. Each ECoG recording probe consists of 17 transistors with channel dimensions of *W* = 15 µm and *L* = 6 µm, along with 8 electrodes measuring 12 × 12 µm². Fig. 6b illustrated the placement of these OECT probes on the rat somatosensory cortex, and the probes exhibit a superior signal-to-noise ratio (SNR) of 44 dB, compared to the SNR of 24.2 dB achieved by the PEDOT:PSS surface electrodes. The SNR calculation involved analyzing the standard deviation (STD) of the highest peak during seizure-like activity and comparing it to the background signal during periods of low biological activity. Notably, the OECTs, benefiting from their local amplification capability, demonstrate higher SNR values than the surface electrodes. This highlights the potential of OECTs as active components for the detection and amplification of biological signals, particularly in the field of active matrix addressing.

Wonryung Lee et al. developed a transparent, ultra-flexible, and active multi-electrode array (MEA) composed of transparent organic electrochemical transistors (OECTs) and transparent Au grid wirings [74]. The circuit diagram for OECT multiplexing was presented in Fig. 6c, demonstrating selective access to the OECT by applying a voltage to the chosen line. Fig. 6d shows the 3 × 5 OECTs array conformally placed on the rat brain, revealing clear identification of cerebral vessels. The Au grid featured a micropattern design that achieved 60% transparency at a wavelength of 475 nm. This transparent and active MEA allowed for the spatial mapping of ECoG electrical signals with amplitudes of ∼0.8 mV from an optogenetic rat, with a spacing of 1 mm between measurement points. Importantly, the MEA exhibited minimal light artifacts, ensuring high-quality signal recording with noise levels (SNR 13 dB) lower than the recorded signals. These findings demonstrate the feasibility of using transparent and active MEA technology, enabling the precise study of neural network systems through direct light stimulation. OECTs are making notable advancements in addressing critical challenges related to neural interfaces, including biodegradability, multi-channel recording, and high-density integration. Mengge Wu et al. proposed an ultra-thin, flexible, and partially biodegradable multichannel neural interface based on a 100-channel OECT. Fig. 6e depicts an effective area of 8 × 8 mm^2^ including 100 OECTs arranged in an array composed of biocompatible materials such as PLGA, gold electrodes, and organic semiconductors like PEDOT:PSS [75]. Fig. 6f demonstrates the OECT arrays gently adhered to the cerebral cortex of 4–6 week-old Sprague Dawley (SD) rats, successfully monitoring enhanced EP signals with an SNR of up to 37 dB on the rat cortical surface. These arrays also facilitate real-time electrocorticographic mapping from 100 channels under various conditions (such as anesthesia, seizures, and electrical stimulation) and spontaneously degrade upon completion of the task, eliminating the need for secondary removal surgery.

OECTs exhibit the promise of stable and functional neural interfaces, but their slow response time limits their ability to capture fast electrophysiological activities. The search for new materials has led to the emergence of a new class of brain-computer interfaces, with graphene standing out due to its high carrier mobility. The carrier mobility values exceeding 10,000 cm^2^ V^–1^ s^–1^, and ideally reach up to 350,000 cm^2^ V^–1^ s^–1^ [76,77]. This exceptional carrier mobility enables graphene solution-gated field-effect transistors (g-SGFETs) to exhibit an incredibly short transient response, surpassing other technologies such as silicon-based transistors (1400 cm^2^ V^–1^ s^–1^) or conducting polymer-based transistors like PEDOT:PSS (0.01 cm^2^ V^–1^ s^–1^) [78,79]. In addition to their fast transient response, g-SGFETs offer several essential features that make them highly attractive for applications inµECoG arrays. These features, including biocompatibility, chemical stability, mechanical flexibility, and a high signal-to-noise ratio, make g-SGFETs well-suited for interfacing with neural tissue to provide accurate and reliable measurements of electrophysiological signals [80], [81], [82].

Nathan Schaefer et al. developed an 8 × 8 gSGFET sensor array with a probe layout consisting of two metallic layers forming the vertical lines of the sensor grid, separated by a polyimide layer in the middle, as illustrated in Fig. 6h [83]. The array consisted of 64 gSGFET sensors with monolayer graphene channels measuring 50 µm × 50 µm in size, and an inter-site separation of 400 µm. Fig. 6i presents a schematic of the addressing and acquisition method of the gSGFET sensor, in which a custom PCB board with two main functional blocks was developed to enable this functionality. One block filtered and amplified the simultaneously acquired AC and DC signals, while the other block selected the line where the bias difference was applied and addressed it using an external switching matrix. In vivo evaluation in Long Evans rats confirmed the fidelity of broadband signal representation, including IR slow oscillations and local field potentials, during multiplexed operation. The signal quality achieved was comparable to that of state-of-the-art commercially available neurosensors.

Ramon Garcia-Cortadella et al. proposed a new approach to frequency division multiplexing (FDM) using graphene solution-gated field-effect transistors (g-SGFETs) [84]. The equivalent circuit of the g-SGFET and the device structure are shown in Fig. 6j, in which the graphene channel is in contact with the electrolyte gate. Potential fluctuations in the environment affect the conductivity of the transistor channel through the gate capacitance. In this approach, the neural signals detected by different graphene active sensors on the array are amplitude-modulated (AM) by different carrier signals, allowing multiple signals to be transmitted over a shared communication channel. As depicted in Fig. 6g, a frequency-division multiplexed array of 4 × 8 graphene sensors was used to record electrical activity in the Long Evans rat cortex. It effectively differentiated between distance-dependent signal amplitude and signal delay when the neural probe was placed on the primary visual cortex. The SG-GFET array successfully recorded CSD events with high fidelity and without distortion, demonstrating the high sensitivity of the gSGFET array to broadband neural signals in vivo.

Blaschke et al. developed flexible 4 × 4 gSGFET arrays on polyimide substrates for in vivo local field potential (LFP) measurements in rat brains [85]. These arrays recorded pre-epileptic interictal spikes induced by bilobalin injection and outperformed Pt electrodes in capturing larger spikes. With a signal-to-noise ratio (SNR) of approximately 62 dB, comparable to state-of-the-art Pt electrodes, the g-SGFETs array shows promise for mapping EEG activity with excellent SNR, offering the potential for in vivo recording of fundamental cellular activity. In another study, Masvidal-Codina et al. investigated flexible epicortical and intracortical arrays of graphene solution-gated field-effect transistors (g-SGFETs) [86]. These arrays demonstrated high-fidelity recording of cortical diffusion suppression (CSD) signals, providing insights into brain function and disease states. The advancements made in these studies highlight the potential of these devices as chronically implantable tools for clinical diagnosis, improving our understanding of the brain and enabling better monitoring of neurological conditions.

## 4 Conclusion and outlook

Accurate recording and localization of neural activity in specific brain regions are crucial for understanding brain neurology and advancing ECoG device applications. To achieve this, enhancing electrode density and channel count in ECoG devices, while considering impedance, mechanical properties, and biocompatibility, is essential. Flexible electronics and microfabrication techniques have opened up opportunities for developing high-density, high-throughput ECoG devices, leading to the emergence of µECoG with sub-millimeter spatial resolution capabilities. In recent years, µECoG has emerged as a pivotal tool in both neurological research and clinical applications. For example, its high spatial resolution facilitates precise localization of epileptic foci, thereby enhancing the targeting accuracy of surgical interventions [87]. Furthermore, µECoG can acquire insightful neural data across expansive coverage areas. This capability is not only vital for unraveling complex neural circuits in the brain [88], but also presents promising prospects in areas such as speech decoding and avatar control in the domain of brain-machine interfaces [13].

Passive multielectrode array (MEA) technology is a well-established solution for µECoG recording with high spatiotemporal resolution. These arrays utilize biocompatible soft materials and employ small, ultra-thin, and porous designs to seamlessly integrate with tissues. Surface modifications can enhance the impedance and biocompatibility of contact electrodes. However, conventional passive MEA arrays require bulky leads as the number of channels increases, limiting their scalability. Active transistor arrays offer a promising solution for high-throughput and high-density µECoG devices by enabling multiplexing and reducing the complexity of lead wires. However, challenges exist regarding signal-to-noise ratio, sampling rate, channel uniformity, and long-term recording capability in current transistor array applications in the µECoG field.

While kilo-scale µECoG devices have achieved high densities of over 25 sites/mm^2^ and have been successfully applied in human brain recording, there is still significant room for improvement [38]. Exploring lead wire arrangements, optimizing connection designs, and developing data acquisition circuits capable of supporting thousands of channels are important for increasing channel count and density in passive MEA arrays. Active transistor arrays offer an alternative approach, but minimizing acquisition noise, optimizing device quality, and electrode uniformity are critical. Long-term recording capability needs further demonstration, especially for transistor arrays that exclude silicon transistors. Interdisciplinary integration is a promising direction for high-throughput and high-density µECoG devices. Optical transparency design enables integration with optogenetics, optical imaging, and neural circuit analysis. Integration with miniature drug delivery designs and electrochemical sensors enables multimodal interfaces. Furthermore, the pursuit of wireless high-throughput µECoG recording, guided by electronic engineering design principles, represents a fascinating area of research. This is because wireless high-throughput µECoG has the potential to transcend the constraints imposed by wired connections, enabling precise recordings of brain regions in subjects engaged in unrestricted activities, thus significantly expanding the potential applications of µECoG. With the recent advancements in ECoG technology, encompassing electrodes, circuits, and data transmission, wireless high-throughput µECoG recording systems are poised for new opportunities [89]. Overall, continuous research and development in lead wire solutions, transistor array optimization, interdisciplinary integration, and wireless recording can drive advancements in this field. We believe that there are substantial opportunities in the pursuit of high-throughput and high-density µECoG devices, enabling precise exploration of brain functions.

## Declaration of competing interest

The authors declare that they have no conflicts of interest in this work.

## References

[1] Parvizi J., Kastner S. (2018). Promises and limitations of human intracranial electroencephalography. Nat. Neurosci..

[2] Michel C.M., Murray M.M. (2012). Towards the utilization of EEG as a brain imaging tool. Neuroimage.

[3] Waldert S. (2016). Invasive vs. non-invasive neuronal signals for brain-machine interfaces: Will one prevail?. Front. Neurosci..

[4] Herff C., Krusienski D.J., Kubben P. (2020). The potential of stereotactic-EEG for brain-computer interfaces: Current progress and future directions. Front. Neurosci..

[5] Li G., Jiang S., Paraskevopoulou S.E. (2018). Optimal referencing for stereo-electroencephalographic (SEEG) recordings. Neuroimage.

[6] Drane D.L., Pedersen N.P., Sabsevitz D.S. (2021). Cognitive and emotional mapping with SEEG. Front. Neurol..

[7] Thakor N.V. (2013). Translating the brain-machine interface. Sci. Transl. Med..

[8] Shokoueinejad M., Park D.-W., Jung Y.H. (2019). Progress in the field of micro-electrocorticography. Micromachines.

[9] Buzsáki G., Anastassiou C.A., Koch C. (2012). The origin of extracellular fields and currents — EEG, ECoG, LFP and spikes. Nat. Rev. Neurosci..

[10] Volkova K., Lebedev M.A., Kaplan A. (2019). Decoding movement from electrocorticographic activity: A review. Front. Neuroinform..

[11] Hermes D., Nguyen M., Winawer J. (2017). Neuronal synchrony and the relation between the blood-oxygen-level dependent response and the local field potential. PLoS Biol..

[12] Shirvalkar P., Prosky J., Chin G. (2023). First-in-human prediction of chronic pain state using intracranial neural biomarkers. Nat. Neurosci..

[13] Metzger S.L., Littlejohn K.T., Silva A.B. (2023). A high-performance neuroprosthesis for speech decoding and avatar control. Nature.

[14] Krishna S., Choudhury A., Keough M.B. (2023). Glioblastoma remodelling of human neural circuits decreases survival. Nature.

[15] Muller L., Hamilton L.S., Edwards E. (2016). Spatial resolution dependence on spectral frequency in human speech cortex electrocorticography. J. Neural Eng..

[16] Yang T., Hakimian S., Schwartz T.H. (2014). Intraoperative ElectroCorticoGraphy (ECog): Indications, techniques, and utility in epilepsy surgery. Epilept. Disord..

[17] Lee M., Shim H.J., Choi C. (2019). Soft high-resolution neural interfacing probes: Materials and design approaches. Nano Lett..

[18] Obien M.E.J., Deligkaris K., Bullmann T. (2015). Revealing neuronal function through microelectrode array recordings. Front. Neurosci..

[19] Alahi M.E.E., Liu Y., Xu Z. (2021). Recent advancement of electrocorticography (ECoG) electrodes for chronic neural recording/stimulation. Mater. Today Commun..

[20] Obidin N., Tasnim F., Dagdeviren C. (2020). The future of neuroimplantable devices: A materials science and regulatory perspective. Adv. Mater..

[21] Zhu M., Wang H., Li S. (2021). Flexible electrodes for in vivo and in vitro electrophysiological signal recording. Adv. Healthc. Mater..

[22] Zhou H.B., Li G., Sun X.N. (2009). Integration of Au nanorods with flexible thin-film microelectrode arrays for improved neural interfaces. J. Microelectromech. Syst..

[23] Sharafkhani N., Kouzani A.Z., Adams S.D. (2022). Neural tissue-microelectrode interaction: brain micromotion, electrical impedance, and flexible microelectrode insertion. J. Neurosci. Methods.

[24] Geddes L.A., Roeder R. (2003). Criteria for the selection of materials for implanted electrodes. Ann. Biomed. Eng..

[25] Wang C., Yokota T., Someya T. (2021). Natural biopolymer-based biocompatible conductors for stretchable bioelectronics. Chem. Rev..

[26] Cho Y.H., Park Y.-G., Kim S. (2021). 3D electrodes for bioelectronics. Adv. Mater..

[27] Geim A.K., Novoselov K.S. (2007). The rise of graphene. Nat. Mater..

[28] Xu B., Pei J., Feng L. (2021). Graphene and graphene-related materials as brain electrodes. J. Mater. Chem. B.

[29] Park D.-W., Schendel A.A., Mikael S. (2014). Graphene-based carbon-layered electrode array technology for neural imaging and optogenetic applications. Nat. Commun..

[30] Donahue M.J., Sanchez-Sanchez A., Inal S. (2020). Tailoring PEDOT properties for applications in bioelectronics. Mater. Sci. Eng.: R: Reports.

[31] Khodagholy D., Gelinas J.N., Thesen T. (2015). NeuroGrid: Recording action potentials from the surface of the brain. Nat. Neurosci..

[32] Schander A., Strokov S., Stemmann H. (2019). A flexible 202-channel epidural ECoG array with PEDOT: PSS coated electrodes for chronic recording of the visual cortex. IEEE Sens. J..

[33] Seo J.-W., Kim K., Seo K.-W. (2020). Artifact-free 2D mapping of neural activity in vivo through transparent gold nanonetwork array. Adv. Funct. Mater..

[34] Li X., Song Y., Xiao G. (2021). PDMS–parylene hybrid, flexible micro-ECoG electrode array for spatiotemporal mapping of epileptic electrophysiological activity from multicortical brain regions. ACS Appl. Bio Mater..

[35] Ryu M., Yang J.H., Ahn Y. (2017). Enhancement of interface characteristics of neural probe based on graphene, ZnO nanowires, and conducting polymer PEDOT. ACS Appl. Mater. Interfaces.

[36] Ganji M., Hossain L., Tanaka A. (2018). Monolithic and scalable Au nanorod substrates improve PEDOT–metal adhesion and stability in neural electrodes. Adv. Healthc. Mater..

[37] Ganji M., Paulk A.C., Yang J.C. (2019). Selective formation of porous pt nanorods for highly electrochemically efficient neural electrode interfaces. Nano Lett..

[38] Tchoe Y., Bourhis A.M., Cleary D.R. (2022). Human brain mapping with multithousand-channel PtNRGrids resolves spatiotemporal dynamics. Sci. Transl. Med..

[39] Song E., Li J., Won S.M. (2020). Materials for flexible bioelectronic systems as chronic neural interfaces. Nat. Mater..

[40] Tybrandt K., Khodagholy D., Dielacher B. (2018). High-density stretchable electrode grids for chronic neural recording. Adv. Mater..

[41] Qi D., Liu Z., Liu Y. (2017). Highly stretchable, compliant, polymeric microelectrode arrays for in vivo electrophysiological interfacing. Adv. Mater..

[42] Yan J., Lu Y., Chen G. (2018). Advances in liquid metals for biomedical applications. Chem. Soc. Rev..

[43] Dong R., Wang L., Hang C. (2021). Printed stretchable liquid metal electrode arrays for in vivo neural recording. Small.

[44] Zhuang Q., Yao K., Wu M. (2023). Wafer-patterned, permeable, and stretchable liquid metal microelectrodes for implantable bioelectronics with chronic biocompatibility. Sci. Adv..

[45] Xu H., Yin L., Liu C. (2018). Recent advances in biointegrated optoelectronic devices. Adv. Mater..

[46] Kaltenbrunner M., Sekitani T., Reeder J. (2013). An ultra-lightweight design for imperceptible plastic electronics. Nature.

[47] Khodagholy D., Gelinas J.N., Zhao Z. (2016). Organic electronics for high-resolution electrocorticography of the human brain. Sci. Adv..

[48] Rockwood D.N., Preda R.C., Yücel T. (2011). Materials fabrication from Bombyx mori silk fibroin. Nat. Protoc..

[49] Patil A.C., Bandla A., Liu Y.-H. (2020). Nontransient silk sandwich for soft, conformal bionic links. Mater. Today.

[50] Toda H., Suzuki T., Sawahata H. (2011). Simultaneous recording of ECoG and intracortical neuronal activity using a flexible multichannel electrode-mesh in visual cortex. Neuroimage.

[51] Kim D.-H., Viventi J., Amsden J.J. (2010). Dissolvable films of silk fibroin for ultrathin conformal bio-integrated electronics. Nat. Mater..

[52] Kaiju T., Inoue M., Hirata M. (2021). High-density mapping of primate digit representations with a 1152-channel µECoG array. J. Neural Eng..

[53] Metzger S.L., Liu J.R., Moses D.A. (2022). Generalizable spelling using a speech neuroprosthesis in an individual with severe limb and vocal paralysis. Nat. Commun..

[54] Moses D.A., Metzger S.L., Liu J.R. (2021). Neuroprosthesis for decoding speech in a paralyzed person with anarthria. N. Engl. J. Med..

[55] Vitale F., Shen W., Driscoll N. (2018). Biomimetic extracellular matrix coatings improve the chronic biocompatibility of microfabricated subdural microelectrode arrays. PLoS One.

[56] Cha G.D., Kang D., Lee J. (2019). Bioresorbable electronic implants: History, materials, fabrication, devices, and clinical applications. Adv. Healthc. Mater..

[57] Yu K.J., Kuzum D., Hwang S.-W. (2016). Bioresorbable silicon electronics for transient spatiotemporal mapping of electrical activity from the cerebral cortex. Nat. Mater..

[58] Sahasrabuddhe K., Khan A.A., Singh A.P. (2021). The Argo: A high channel count recording system for neural recording in vivo. J. Neural Eng..

[59] Zhao E.T., Hull J.M., Mintz Hemed N. (2023). A CMOS-based highly scalable flexible neural electrode interface. Sci. Adv..

[60] Viventi J., Kim D.-H., Vigeland L. (2011). Flexible, foldable, actively multiplexed, high-density electrode array for mapping brain activity in vivo. Nat. Neurosci..

[61] Escabí M.A., Read H.L., Viventi J. (2014). A high-density, high-channel count, multiplexed µECoG array for auditory-cortex recordings. J. Neurophysiol..

[62] Fang H., Zhao J., Ki J.Yu (2016). Ultrathin, transferred layers of thermally grown silicon dioxide as biofluid barriers for biointegrated flexible electronic systems. Proc. Natl. Acad. Sci..

[63] Fang H., Yu K.J., Gloschat C. (2017). Capacitively coupled arrays of multiplexed flexible silicon transistors for long-term cardiac electrophysiology. Nat. Biomed. Eng..

[64] Chiang C.-H., Won S.M., Orsborn A.L. (2020). Development of a neural interface for high-definition, long-term recording in rodents and nonhuman primates. Sci. Transl. Med..

[65] Seo K.J., Hill M., Ryu J. (2023). A soft, high-density neuroelectronic array. npj Flexible Electron..

[66] Shaik F.A., Ihida S., Ikeuchi Y. (2020). TFT sensor array for real-time cellular characterization, stimulation, impedance measurement and optical imaging of in-vitro neural cells. Biosens. Bioelectron..

[67] Zhang F., Zhang L., Xia J. (2023). Multimodal electrocorticogram active electrode array based on zinc oxide-thin film transistors. Adv. Sci..

[68] Huang X., Londoño-Ramírez H., Ballini M. (2022). 2022 IEEE International Solid-State Circuits Conference (ISSCC).

[69] Huang X., Londoño-Ramírez H., Ballini M. (2022). Actively multiplexed µECoG brain implant system with incremental-ΔΣ ADCs employing bulk-DACs. IEEE J. Solid-State Circuits.

[70] Owens R.M., Malliaras G.G. (2010). Organic electronics at the interface with biology. MRS Bull..

[71] Rivnay J., Inal S., Salleo A. (2018). Organic electrochemical transistors. Nat. Rev. Mater..

[72] Kittlesen G.P., White H.S., Wrighton M.S. (1984). Chemical derivatization of microelectrode arrays by oxidation of pyrrole and N-methylpyrrole: Fabrication of molecule-based electronic devices. J. Am. Chem. Soc..

[73] Khodagholy D., Doublet T., Quilichini P. (2013). In vivo recordings of brain activity using organic transistors. Nat. Commun..

[74] Lee W., Kim D., Matsuhisa N. (2017). Transparent, conformable, active multielectrode array using organic electrochemical transistors. Proc. Natl. Acad. Sci..

[75] Wu M., Yao K., Huang N. (2023). Ultrathin, soft, bioresorbable organic electrochemical transistors for transient spatiotemporal mapping of brain activity. Adv. Sci..

[76] Gosling J.H., Makarovsky O., Wang F. (2021). Universal mobility characteristics of graphene originating from charge scattering by ionised impurities. Commun. Phys..

[77] Banszerus L., Schmitz M., Engels S. (2015). Ultrahigh-mobility graphene devices from chemical vapor deposition on reusable copper. Sci. Adv..

[78] Ng H.K., Xiang D., Suwardi A. (2022). Improving carrier mobility in two-dimensional semiconductors with rippled materials. Nat. Electron..

[79] Stavrinidou E., Leleux P., Rajaona H. (2013). Direct measurement of ion mobility in a conducting polymer. Adv. Mater..

[80] Bullock C.J., Bussy C. (2019). Biocompatibility considerations in the design of graphene biomedical materials. Adv. Mater. Interfaces.

[81] Lee C., Wei X., Kysar J.W. (2008). Measurement of the elastic properties and intrinsic strength of monolayer graphene. Science.

[82] Kuzum D., Takano H., Shim E. (2014). Transparent and flexible low noise graphene electrodes for simultaneous electrophysiology and neuroimaging. Nat. Commun..

[83] Schaefer N., Garcia-Cortadella R., Martínez-Aguilar J. (2020). Multiplexed neural sensor array of graphene solution-gated field-effect transistors. 2D Mater..

[84] Garcia-Cortadella R., Schafer N., Cisneros-Fernandez J. (2020). Switchless multiplexing of graphene active sensor arrays for brain mapping. Nano Lett..

[85] Blaschke B.M., Tort-Colet N., Guimerà-Brunet A. (2017). Mapping brain activity with flexible graphene micro-transistors. 2D Mater.

[86] Masvidal-Codina E., Illa X., Dasilva M. (2019). High-resolution mapping of infraslow cortical brain activity enabled by graphene microtransistors. Nat. Mater..

[87] Barth K.J., Sun J., Chiang C.-H. (2023). Flexible, high-resolution cortical arrays with large coverage capture microscale high-frequency oscillations in patients with epilepsy. Epilepsia.

[88] Nakahara K., Adachi K., Kawasaki K. (2016). Associative-memory representations emerge as shared spatial patterns of theta activity spanning the primate temporal cortex. Nat. Commun..

[89] Ji B., Liang Z., Yuan X. (2022). Recent advances in wireless epicortical and intracortical neuronal recording systems. Sci. China Inf. Sci..

[90] Ledochowitsch P., Félus R.J., Gibboni R.R. (2011). 2011 IEEE 24th International Conference on Micro Electro Mechanical Systems.

[91] Ledochowitsch P., Koralek A.C., Moses D. (2013). 2013 35th Annual International Conference of the IEEE Engineering in Medicine and Biology Society (EMBC).

